# Age- and Disease-Dependent HERV-W Envelope Allelic Variation in Brain: Association with Neuroimmune Gene Expression

**DOI:** 10.1371/journal.pone.0019176

**Published:** 2011-04-29

**Authors:** Rakesh K. Bhat, Kristofor K. Ellestad, B. Matt Wheatley, Rene Warren, Robert A. Holt, Christopher Power

**Affiliations:** 1 Division of Neurology, University of Alberta, Edmonton, Alberta, Canada; 2 Division of Neurosurgery, University of Alberta, Edmonton, Alberta, Canada; 3 British Columbia Cancer Agency, Genome Science Centre, Vancouver, British Columbia, Canada; National Institutes of Health, United States of America

## Abstract

**Background:**

The glycoprotein, Syncytin-1, is encoded by a human endogenous retrovirus (HERV)-W *env* gene and is capable of inducing neuroinflammation. The specific allele(s) responsible for Syncytin-1 expression in the brain is uncertain. Herein, HERV-W *env* diversity together with Syncytin-1 abundance and host immune gene profiles were examined in the nervous system using a multiplatform approach.

**Results:**

HERV-W *env* sequences were encoded by multiple chromosomal encoding loci in primary human neurons compared with less chromosomal diversity in astrocytes and microglia (*p*<0.05). HERV-W *env* RNA sequences cloned from brains of patients with systemic or neurologic diseases were principally derived from chromosomal locus 7q21.2. Within the same specimens, HERV-W *env* transcript levels were correlated with the expression of multiple proinflammatory genes (*p*<0.05). Deep sequencing of brain transcriptomes disclosed the *env* transcripts to be the most abundant HERV-W transcripts, showing greater expression in fetal compared with healthy adult brain specimens. Syncytin-1's expression in healthy brain specimens was derived from multiple encoding loci and linked to distinct immune and developmental gene profiles.

**Conclusions:**

Syncytin-1 expression in the brain during disease was associated with neuroinflammation and was principally encoded by a full length provirus. The present studies also highlighted the diversity in HERV gene expression within the brain and reinforce the potential contributions of HERV expression to neuroinflammatory diseases.

## Introduction

Human endogenous retroviruses (HERVs) entered the human genome through repeated integration events over the past 2–30 million years [1], [2] and have been implicated in both health and disease including neuropsychiatric disorders such as multiple sclerosis (MS) and schizophrenia [3], [4]. In some instances, the complete provirus is initially integrated but incomplete retroviral sequences are also integrated, albeit at different loci on multiple chromosomes resulting in the presence of paralogs of original ancestral viral genes throughout [5], [6], [7], [8], [9]. Depending on the viral sequence, proximal host genes' expression and function may be altered, particularly if the HERV's long terminal repeat (LTR) containing a promoter and enhancer is integrated near a susceptible host gene. In less frequent circumstances, a HERV gene may be expressed as a partial or complete open reading frame (ORF), which can result in translation of the corresponding protein [10], [11].

Molecular diversity within envelope (*env*) sequences of retroviruses and other viruses influence several cellular functions including susceptibility to infection, immune responses, growth and survival [12]. The HERV-W *env*-encoded glycoprotein, Syncytin-1, is expressed during placental development and is thought to be involved in syncytiotrophoblast maturation [3], [13], [14], [15]. It is widely assumed that the *env* transcript responsible for encoding Syncytin-1 is derived from the HERV-W full length provirus located on chromosome 7q21.2, termed *ERVWE1* [16], [17]. HERV-W *env* transcripts and the corresponding protein, Syncytin-1, have also been reported to be expressed in glial cells within demyelinating lesions in brains from patients diagnosed with MS [4], [18]. Over-expression of Syncytin-1 in astrocytes causes an unfolded protein response resulting in endoplasmic reticulum stress; there is also ensuing neuroinflammation with concurrent induction of interleukin-1β and NOS2 [19]. The extent of molecular diversity within Syncytin-1 encoding sequences due to different HERV-W *env* alleles is unknown although multiple HERV-W *env* integration loci are recognized in the human genome [20], [21].

Given these circumstances, the working hypothesis for the present studies was: HERV-W *env* sequences responsible for encoding Syncytin-1 in the brain might be derived from multiple alleles and associated with neuroimmune gene expression depending on the host's age and health status. To understand the extent and potential interactions of HERV-W *env* diversity with host neuroimmune responses, an approach was adopted in which sequencing was performed with stringent assignment of HERV-W *env* paralogs to specific encoding loci; in addition, deep sequencing of brain specimens was also applied. The present studies revealed HERV-W *env* sequences in the brain were encoded by multiple alleles but among patients with neurologic or systemic disease, the principal encoding locus was 7q21.2/*ERVWE1*. Moreover, HERV-W *env* expression was correlated with inflammatory gene expression in disease but was associated with a distinct innate immune and developmental profile in healthy brain specimens.

## Results

### HERV-W *env* paralog sequences

**Figure 1:**
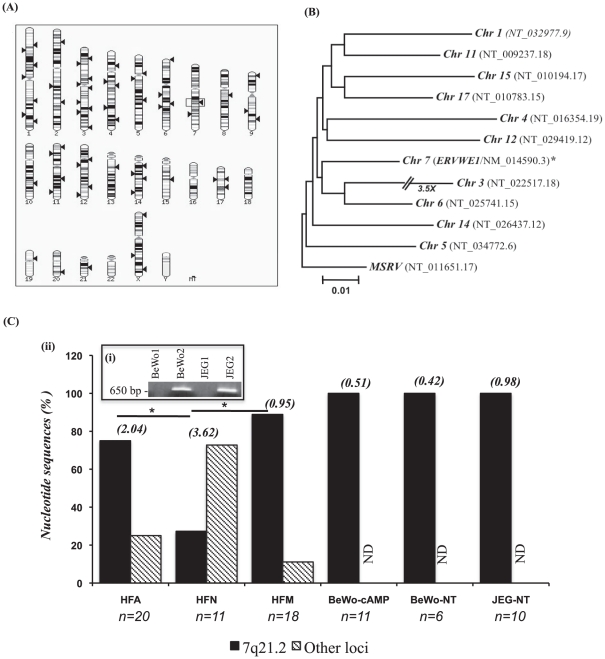
HERV-W *env* sequence mapping and detection. The human genome contains several endogenous retroviral envelope sequences similar to *ERVWE1*. (**A**) Chromosomal loci (arrows) depicting sequences similar to *ERVWE1 (accession No.* NM_014590). The genomic location of the *ERVWE1* locus on chromosome 7 is indicated with a box. (**B**) Phylogenetic analysis of HERV-W *env* gene at 12 different putative gene loci; the dendrogram was constructed based on DNA sequence by the Neighbor-Joining method using the MEGA4 program. The tree is rooted to the MSRV prototype *env* sequence, which was used as an out-group gene sequence. (**C**) Amplification and cloning of the 650 bp region of HERV-W *env* gene: (**i**) ethidium bromide-stained agarose gel showing HERV-W *env* gene amplicons JEG and BeWo cell lines, (**ii**) frequency of HERV-W *env* cDNA sequences cloned from human fetal astrocytes (HFA), neurons (HFN), microglia (HFM) and immortalized cell lines. In primary cells, HERV-W *env* sequences were derived from the 7q21.2 (ERVWE1) locus as well as other loci, while all HERV-W *env* sequences from immortalized cell lines appeared to be encoded by 7q21.2. The values in parenthesis indicate percent diversity among clones for each patient relative to ERVWE1. (ND = none detected).

HERV-W *env* sequences are comprised of multiple paralogs, derived from different chromosomal loci within the human genome (**Figure 1A**). The prototypic HERV-W *env* sequence (*ERVWE1*; accession no.: NM_014590) is located at chromosomal locus 7q21.2 and is expressed as the full length Syncytin-1 open reading frame. This sequence was compared to similar sequences within the human genome including expressed sequence tags; the retrieved sequences exhibited sequence similarity of greater than 90% to *ERVWE1* (**Figure 1B**) but none encoded the full open reading frames homologous across the full length of ERVWE1. These sequences were aligned, revealing clustering with the *ERVWE1* sequence. Using PCR primers targeting the most conserved regions that flanked a region of high sequence diversity within this latter group of sequences (**Figure S1**), HERV-W *env* paralogs were amplified spanning the surface unit-transmembrane junction domain from primary human astrocytes, microglia and neurons together with placenta-derived cell lines (**Figure 1C**). The resulting ∼650 bp amplicon (**Figure 1Ci**) containing this heterogenous region of HERV-W *env* gene was cloned, sequenced (≥11 cloned sequences per specimen) and aligned with established HERV-W *env* sequences. There was substantial HERV-W *env* sequence diversity within the primary neuron (HFN) derived sequences compared to primary astrocytes (HFAs) and primary microglia (HFMs) (p<0.05), indicative of multiple encoding loci (**Figure 1Cii**). Conversely, the placental cell line-derived (BeWo, JEG) sequences uniformly showed high sequence similarity to the *ERVWE1* sequence. These findings underscored the variation in HERV-W *env* transcripts depending on the cellular source of RNA being examined.

### HERV-W *env* and host gene expression in autopsied brain white matter

**Figure 2:**
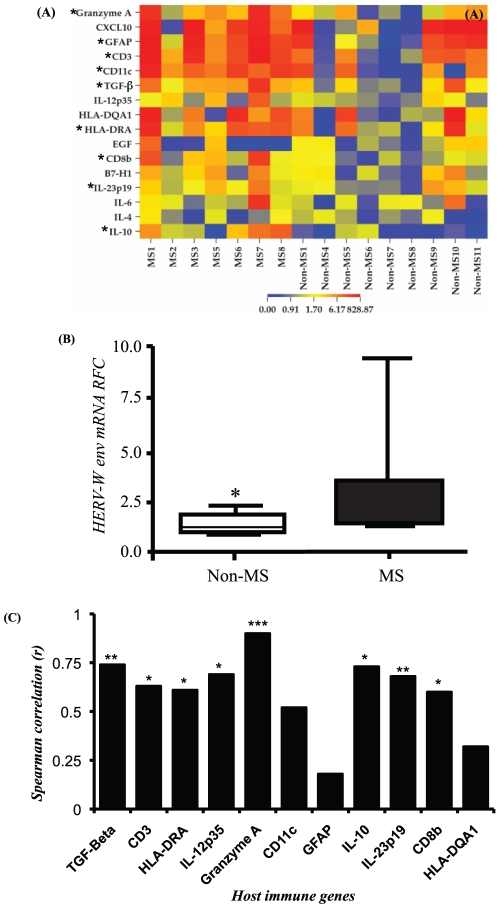
HERV-W *env* and immune gene expression in brain white matter. (**A**) A heatmap showing relative expression of host immune genes relevant to neuroinflammation in MS and non-MS white matter samples, generated based on real time RT-PCR studies (* indicates statistically up- or down regulated genes). (**B**) Median HERV-W *env* transcript levels in non-MS and MS autopsied white matter specimens showing increased levels in MS brain-derived samples. (**C**) Regression analyses of host immune gene with HERV-W *env* transcript abundance. Transcripts expressed as relative fold change to the house keeping gene, GAPDH. (Spearman r; Mann-Whitney, *U* test, *p<0.05, **p<0.005, ***p<0.0005).

Previous studies have reported pro-inflammatory genes are up-regulated in the brains of patients with MS. Examination of immune gene expression among MS and non-MS brain white matter disclosed inflammatory gene transcripts were increased in the MS compared with non-MS specimens (**Figure 2A**). Furthermore, HERV-W *env* transcript levels were also significantly increased in MS brains (**Figure 2B**). The latter findings were further emphasized by significant correlations between HERV-W *env* expression and *granzyme A*, *CD3ε*, *CD8β*, *TGFβ*, *IL-12 and IL-23* transcript levels (**Figure 2C**). These observations recapitulated earlier reports of increased Syncytin-1 expression in MS brains but also indicated a robust association between its expression and neuroinflammation.

### HERV-W *env* transcript molecular diversity in brain

**Figure 3:**
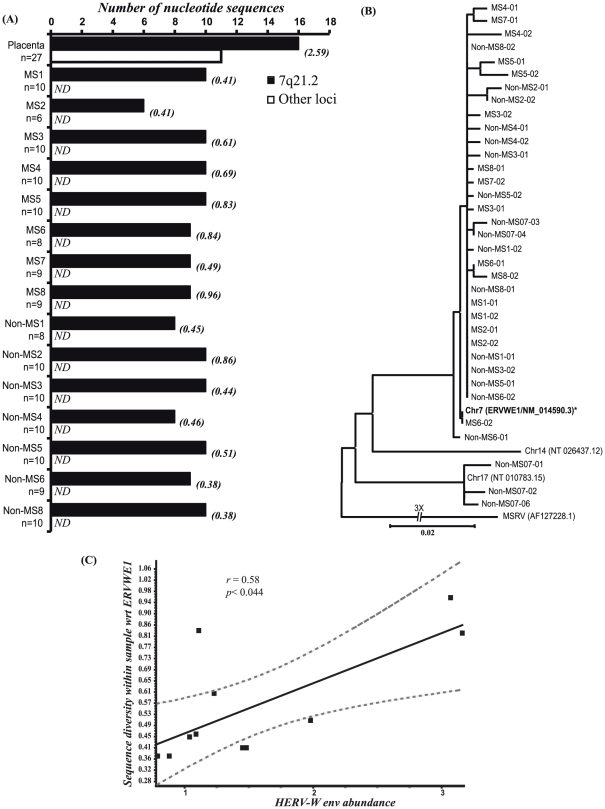
HERV-W *env* sequence analyses from autopsied brain white matter. (**A**) Chromosomal loci encoding HERV-W *env* sequences in MS and non-MS samples showed 7q21.2 was the predominant locus in MS and non-MS samples, while placental clones were derived from multiple chromosomal locations. Values in parenthesis indicate percent diversity among clones for each patient relative to ERVWE1. (**B**) Phylogenetic analysis of HERV-W *env* sequences from MS and non-MS samples, based on DNA sequence by the neighbor-joining method. The tree was rooted to MSRV prototype *env* sequence, which was used as an out-group gene sequence. (**C**) Correlation between sequence diversity within samples with respect to ERVWE1 and HERV-W *env* abundance in MS and non-MS samples. (ND = none detected).

The HERV-W *env* sequences encompassing the 650 bp region containing the surface unit-transmembrane junction were analyzed in brain-derived cDNA from MS and non-MS patients. Alignment of sequences showed that cloned HERV-W *env* brain-derived sequences from MS and non-MS patients were most closely aligned with the chromosome locus at 7q21.2 (*ERVWE1*) sequence (**Figure 3A**), in contrast to placenta-derived sequences, which appeared to be encoded by multiple transcripts. Indeed, within multiple brain-derived sequences (≥6) obtained per patient, there was minimal molecular diversity relative the *ERVWE1* sequence (0.3–0.94%), regardless of the individual patient's neurologic diagnosis. To examine the phylogenetic relationships of representative sequences obtained from different brain-derived samples, a rooted neighbor-joining tree was constructed showing a clustering with the *ERVWE1* sequence with clear sequence segregation (**Figure 3B**) from the MSRV *env* sequence, another prototype HERV-W sequence [17]. In fact, all the brain-derived sequences were derived from a single branch of the tree. There was no definitive clustering of sequences from MS versus non-MS patients. Nonetheless, analysis of molecular diversity within the MS and non-MS clones showed a significant correlation between HERV-W *env* transcript levels and the intra-patient cloned sequence diversity (**Figure 3C**). This analysis was extended by calculating the relative synonymous (K_s_) and non-synonymous (K_a_) rates within subjects, which did not disclose differences between clinical groups (**Table S1**). Thus, in adult brain white matter autopsied samples, all detected HERV-W *env* sequences were derived from the *ERVWE1* locus on chromosome 7q21.2 and exhibited restricted molecular diversity.

### HERV-W *env* deep sequencing analysis of healthy adult and fetal brains

**Figure 4:**
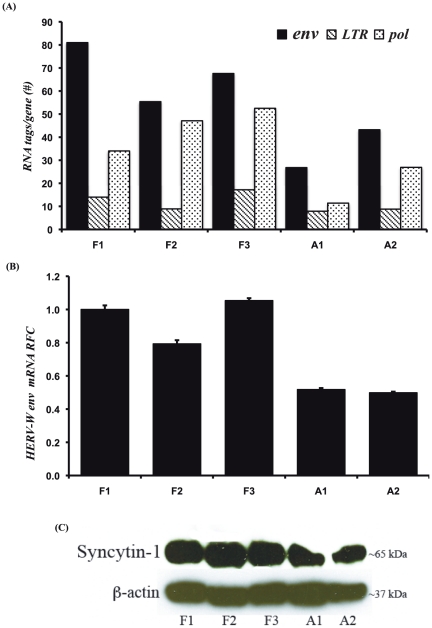
HERV-W *env* RNA and Syncytin-1 abundance in healthy human fetal and adult brain samples. (**A**) Deep sequencing revealed HERV-W and related -*env*, -*LTR* and -*pol* RNA tags in fetal and adult samples, although the frequency of *env* tags was highest in all specimens. (**B**) Transcript levels of HERV-W *env* gene normalized to GAPDH, were highest in fetal brain samples. (**C**) Western blot analysis disclosed up-regulation of Syncytin-1 immunoreactivity in fetal samples relative to β-actin expression.

The above data highlighted the expression of HERV-W *env* in adult human brain in the context of disease, but to gain more insight into its expression, three fetal (F1-3) and two adult (A1-2) brain samples were assessed by deep sequencing, which yielded multiple HERV-W RNA-derived sequence tags. These sequence tags were searched against the human genome and EST database by BLAST [22]. HERV-W *env* was the most frequently detected sequence tag within the HERV-W family among all patient samples followed by *pol* and the LTR but gag was not consistently detected (**Figure 4A**); HERV-W *env* transcripts were most abundant among the fetal brain samples. Analysis of HERV-W *env* transcript abundance in the same samples by real time RT-PCR disclosed a similar profile with augmented expression in fetal brains (**Figure 4B**). Moreover, Syncytin-1 immunoreactivity on Western blot also showed increased expression in the fetal brains (**Figure 4C**), indicating that HERV-W *env* and Syncytin-1 abundance might be developmentally-related.

### HERV-W *env* diversity and host gene expression

**Figure 5:**
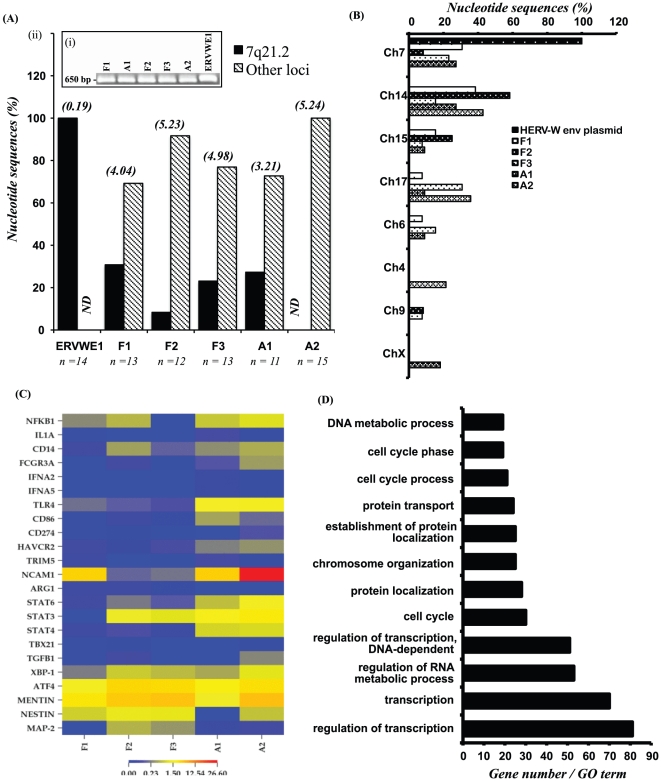
HERV-W *env* sequence analyses in healthy brain specimens. (**A**) (**i**) Ethidium-stained agarose gel of amplicons after second PCR step from fetal (F) and adult (A) brain samples. (**ii**) Relative clonal frequency of transcriptionally active HERV-W *env* sequences in fetal and adult brain samples showing encoding loci to be 7q21.2 and other sites. Diversity relative to ERVWE1 is displayed in parenthesis. (**B**) Chromosomal loci distribution of transcriptionally active HERV-W *env* sequences in fetal and adult brain samples. (**C**) Heatmap of gene expression was generated from RNA tags obtained by deep sequencing analysis by selecting a set of host genes relevant to neuroinflammation and associated with HERV-W *env* abundance in the sample. Over-expressed genes are shown in red and under-expressed in blue. (**D**) Host genes with expression profiles highly correlated with *HERV-W env* tag abundance (r^2^≥0.75) were analyzed using DAVID tools (http://david.abcc.ncifcrf.gov/) for enriched gene ontology (GO) terms. Genes related to transcriptional activity and regulations were strongly associated with HERV-W *env* expression. (ND = none detected).

The HERV-W *env* sequence encoding the surface unit-transmembrane region (650bp) was amplified (**Figure 5Ai**), cloned (≥11 per specimen) from the fetal and adult brain (non-disease) specimens and subsequently aligned (**Figure 5Aii**). Overall, non-*ERVWE1*-derived sequences were more frequently detected in the present specimens from healthy subjects; in fact, *ERVWE1*-derived sequences were evident in only one adult (A1) but in all fetal samples (**Figure 5Aii**). To confirm that the observed molecular diversity was not a consequence of *Taq* polymerase introduced mutations, the plasmid containing the *ERVWE1*/Syncytin-1 gene was used as control in all PCR assays and disclosed minimal sequence variability (0.02% among 14 cloned sequences) although sequence diversity within the clinical samples ranged from 4.09 to 5.35% depending on the individual sample (**Figure 5Aii**). Sequence alignments and phylogenetic analyses of the HERV-W *env* sequences showed that brain-derived sequences grouped with multiple HERV-W *env* sequences located on different chromosomes (**Figure S2**). Among the non-ERVWE1-derived sequences, chromosomes 14 and 15 (**Figure 5B**) were the most active sites of HERV-W *env* transcription while the X chromosome was the least active. These observations emphasized the multiplicity of encoding loci for potential HERV-W *env* alleles, which depended on age and health status of the subject.

Given the association of Syncytin-1 with neuroinflammation (Figure 1), select immune genes, based on transcript-derived tag frequency were interrogated for their expression levels in all five healthy brain specimens (**Figure 5C**). The resulting heat map showed there was differential expression of immune genes distinguishing fetal from adult brains and this profile was interpreted in relation to HERV-W *env* transcript abundance. Of particular interest was the relative reduction in *STAT4* and *TLR4* with a concurrent increase in *nestin* in the fetal samples (**Figure 5C**). Vimentin was consistently expressed across all samples in keeping with HERV-W *env's* predominant expression in astrocytes. Of note, several lymphocyte markers including *CD3*, *CD4* and *CD8* were not detected in the fetal samples and were infrequently present in the adult samples (data not shown). Bioinformatic analyses were also performed to investigate the functional aspects of differentially expressed host genes in all brain specimens (**Figure 5C**); all RNA-derived gene tags with recognized identity (specific accession numbers) and of which at least 2 tags were detected per sample were included in this analysis. The expression level of each tag in the fetal and adult samples was correlated (r^2^) with the corresponding expression level of HERV-W *env* in each of the samples. Genes that showed a high correlation (r^2^>0.75) with HERV-W *env* levels were selected for gene ontology analysis. Gene ontology (GO) term analysis of these genes revealed that the majority of the highly correlated tags were associated with processes in which transcriptional regulation participated (**Figure 5D**). These latter observations pointed to an association of HERV-W *env* with specific immune genes but also highlighted its link to processes implicated in development and transcriptional regulation.

## Discussion

HERVs entered the human genome millions of years ago possibly through zoonotic infections from other species, as observed for HIV-1 and -2 and HTLV-1 and -2 but have continued to spread within the genome over time [6], [23], [24]. It is likely that whatever limited molecular diversity observed in HERV-W *env* is a consequence of HERV proliferation within the genome, perhaps as transposable elements but not likely as repeated super-infections; hence, the diversity observed herein was limited despite the multiplicity of genomic loci involved. Despite HERV-W *env* propagation throughout the genome, the predominant locus responsible for Syncytin-1 in the brain during disease was *7q21.2* based on the present studies, likely because the requisite domains for efficient ORF transcription and ensuing translation are expressed within the full-length provirus. The preservation of Syncytin-1 in the human genome, as a retroviral protein might reflect its relative lack of immunosuppressive functions, unlike other retroviral envelope proteins, together with its capacity to induce host immune functions, perhaps with beneficial actions in the placenta in which it is expressed in healthy circumstances.

Comparative analyses of the transcriptomes using rigorous deep sequencing methods from fetal and adult brains have not been performed previously, particularly with a focus on a HERV gene. HERV-W *env* expression indicated by RNA-derived tag frequency, transcript-derived amplicons and Syncytin-1 immunoblotting showed increased expression in the fetal brains. Moreover, for all healthy brain samples, HERV-W *env* transcripts were largely encoded by non-7q21 loci. This finding might reflect a lack of adaptive immune cells and proteins in these brains, unlike the diseased brains, which encoded HERV-W *env* exclusively from 7q21.2. Analyses of all tags revealed that HERV-W *env*-like sequences appeared to be derived from chromosome 7q21.2 but because of the short length of the present tags, a refined assignment of chromosomal locus of origin could not be performed. Tags corresponding to the CD14 sequence, a marker of myeloid cells as well as TLR4, another surrogate marker of myeloid cells, were present in all samples, emphasizing the conservation of innate immunity in the brain, unlike adaptive immune cells (CD3- or CD20-positive cells). Predictably, nestin, a marker of stem cells, was increased in the fetal samples; vimentin was consistently expressed in all samples (**Figure 5C**) while glial fibrillary acidic protein-encoding tags were minimally detected. Thus, the immune milieu in the non-diseased brain is defined largely by homeostatic innate immune gene expression without astrocyte or adaptive immune reactivity, which occurs in response to injury or infection.

The burgeoning annotation of the human genome over the past decade has resulted in greater recognition of the diversity of retroelements; the multiplicity of potential HERV-W *env* encoding regions also underscores the growing complexity of HERV biology. In fact, this increasing density of HERV sequence identification stems from the dynamic nature of retroelements and their capacity to move within the host genome. A question for future studies will be the delineation of the determinant (inflammatory and degenerative) mechanisms by which human retroelements are activated and transpose to other genomic loci in relation to disease. Herein, the coding locus for HERV-W *env* in brain varied depending on the tissue specimens; in particular, autopsied specimens displayed only sequences derived from *ERVWE1* but the sources of HERV-W *env* sequences were more heterogenous in non-diseased brains, perhaps reflecting age or underlying disease status. Both MS and non-MS patients died of diseases while the fetal and adult non-diseased brains were apparently free of inflammatory disease processes, perhaps resulting in differential promoter activation with disease (or age). Another explanation for the paucity of molecular diversity in autopsied white matter-derived HERV-W *env* sequences might lie in the cellular composition of tissues used herein: The healthy brain specimens studied were comprised of white matter and cortex; moreover, neurons exhibited diverse HERV-W *env* transcripts in contrast to glial cells, which displayed less sequence heterogeneity, perhaps reflecting the findings in the present white matter samples. Alternatively, a skewed population of transcripts could emerge if the rates of RNA degradation differed between transcripts, which could distinguish diseased from non-diseased samples due to the fact that diseased samples were collected at autopsy (several hours after death) while non-diseased samples were flash frozen immediately after collection or surgical resection. For example, while the *bona fide* Syncytin-1 (*ERVWE1*) transcript derived from chromosome 7q21.2 is well known to encode protein, the putative paralogs identified in this study might not express protein and thus, could be subject to an increased rate of degradation due to nonsense-mediated RNA decay [25], [26], [27]. Given our increasing recognition of the biological role of non-coding RNA, the expressed HERV-W *env* sequences originating from loci, other than 7q21.2, might be involved in maintaining the euchromatic status of the regions of the genome from which they are expressed. Another intriguing possibility is that they may be involved in the regulation of Syncytin-1 expression, acting as decoys for miRNAs, which target HERV-W *env* transcripts [28]. Furthermore, these concepts may apply broadly to many of the endogenous non-protein encoding retroelements found widely throughout eukaryotic genomes. These results, taken together, provide evidence that HERV-W *env* alleles are expressed by multiple encoding loci and also highlight the link between Syncytin-1 expression and its encoding RNA with host gene expression in the brain.

### Conclusions

In the present studies, HERV-W *env* encoding alleles were principally derived from the 7q21.2/ERVWE1 locus in autopsied brain samples from both MS and non-MS persons; while in primary neural cells and healthy brain specimens (fetal and adult), multiple loci encoded HERV-W *env* alleles. Moreover, HERV-W *env* RNA abundance was highly correlated with pro-inflammatory gene expression in diseased brains; while in healthy brains, there was an association with transcriptional function and innate immunity. These studies represent the first multiplatform analysis of the brain transcriptome focusing on a HERV in relation to the host neuroimmune response. In addition, the current findings also highlight the potential interactions between different HERV encoding sequences with brain development and disease.

## Materials and Methods

### Standard Protocol Approvals, informed consents and ethic statements

The use of autopsied or surgical brain tissue is part of an ongoing research protocol number 2291 approved by the University of Alberta Human Research Ethics Board (Biomedical) and written informed consents were signed before or at the collection time. Human fetal tissues were obtained from 15–19 week aborted fetuses with written consent approved under the protocol 1420 by the University of Alberta Human Research Ethics Board (Biomedical). The protocols for obtaining post-mortem brain samples comply with all federal and institutional guidelines with special respect for the confidentiality of the donor's identity.

### Human brain tissue and cultured cells

For HERV-W *env* sequence analysis, RNA was extracted from autopsied brains of MS subjects [n = 8; median age, 56 yr; males (n = 4); median EDSS, 8.5, secondary progressive (n = 6), progressive relapsing (n = 2)], non-MS subjects [n = 8; median age, 60 yr; males (n = 5); sepsis (n = 2), myocardial infarction (n = 1), systemic cancer (n = 2), HIV/AIDS (n = 3)] as well as healthy fetal [n = 3; 14–18 wk] and adult brain surgical specimens [n = 2; 15–25 yr] mesial temporal sclerosis, remote from the epileptogenic focus], human placenta, primary and immortalized cell lines, as described elsewhere [29], [30].

### Deep sequencing and analyses of brain transcriptome

Ten micrograms of total RNA was treated with Promega RNase-free DNase in a final volume of 50 µL. The reaction was incubated for 30 min at 37°C and stopped by incubation for 10 min at 65°C. Double-strand cDNA (ds-cDNA) was generated using the ds-cDNA synthesis kit (Invitrogen) according to the manufacturer's instructions. The resulting ds-cDNA was cleaned and single end tag (SET) sequencing was performed using the Illumina Genome Analyzer per the manufacturer's instructions. Short read sequences obtained from the Illumina Genome Analyzer were mapped to the reference HERV mRNAs from NCBI database.

### RT-PCR analysis

For amplification of the HERV-W *env* gene-related paralogs, total RNA was extracted from brain white matter collected at autopsy from age-matched MS and non-MS patients, as well as from healthy fetal and adult subjects as described previously [19], [31], [32]. 700–800 ng of RNA was reverse-transcribed with SuperScript II (Invitrogen) as previously reported [33], [34], [35]. To increase the amplification yield and specificity of HERV-W *env* and predicted paralogs, different sets of nested PCR primers were designed together conserving regions identified in alignments of HERV-W *env* and the predicted paralogs. The primer sets that resulted in the maximum and correct amplicon yield were used in all further analyses. The size and identity of the PCR-amplified product was confirmed on agarose gel as well as by dideoxy sequencing.

### Cloning, sequencing and alignment of HERV-W *env* sequences

Complementary DNA derived from the RNA of the cells and samples was subjected to nested PCR using a High-Fidelity DNA Polymerase (Phusion, NEB) and varied combinations of HERV-W *env* gene specific primer pairs spanning over the surface unit-transmembrane (SU-TM) region. Sense1: GATGGATCCAAAACTACATGAAACCT and anti-sense1: CGTGACTCGAGC TGCTTCCTGC as outer primers and sense2: GATGGATCCAGCCTATTTAATACCAC and anti-sense2: CGTGACTCGAGAGTTAAGTTGATCTTGCAA as inner pair of primers to amplify a 650 bp fragment spanning the surface-transmembrane region of the envelope genes, (see **Table S2** in the supplemental material for additional oligonucleotide primer sequences). The amplified fragments were cloned into the pGEM-T Easy vector (Promega) or pBlueScript SK II (+) vector (Fermentas) and sequenced with double coverage with dideoxy terminators (ABI BigDye- Applied Biosystems or DYEnamic ET Terminator Dye- Amersham Biosciences). Sequencing data generated on Applied Biosystems 3730 DNA Analyzer with 3739 Data Collection Software v3.0 were analyzed using Sequence Analysis Software 5.2. KB Basecaller v1.2, which assigns a base and quality value (QV) to each peak was used in the analysis protocol [36], [37]. A total of 6–27 clones were sequenced for each sample to obtain a representative measurement for the diversity within the viral gene. The nucleotide sequences obtained were confirmed by comparing with the NCBI database and the sequences were trimmed to include only the SU-TM region of interest. The sequence traces were visually examined and analyzed by FinchTV V1.4.0 software (**Geospiza Inc.**) to confirm the nucleotide base-calling, and alignment and Neighbor-Joining analysis was performed using with Molecular Evolutionary Genetics Analysis software, **MEGA4** [38]. The sequences were first aligned against the Syncytin-1 (*ERVWE1*) proviral *env* gene with Clustal W program (European Bioinformatics Institute, Hinxton, UK). A dendrogram was constructed thereafter using the MEGA4. The sequence alignments used to construct the dendrogram are provided (Figure S1). All sequences were also checked for the closest relatives by BLAST search (http://blast.ncbi.nlm.nih.gov/Blast.cgi) at the nucleotide level against the whole human genome and known expressed sequenced tags (EST). To obtain an estimate of nucleotide diversity in Syncytin-1 gene transcripts in brain, the normalized numbers of variant sites (θ) was calculated by dividing the number of observed nucleotide changes by the length (base pairs) of the sequence and the correcting for the sample size (n) as described [39].

### Assignment of nucleotide sequences

The assembled sequences were analyzed further to exclude ambiguities. The nucleotide sequences were subjected to a similarity search using BLASTN against the entire human genome and the known EST database. A blast similarity score (Max identity) over a query length of 580 or higher bps of greater than 97% similarity was considered as a match for the purpose of assigning the locus of the transcript's origin. The chromosomal/transcript assignment was based on the sequence alignment of the cloned sequences against the entire human genome and EST, which overlapped with the HERV-W *env* sequence. Based on this approach, all nucleotide sequences were assigned to specific loci in the genome [40]. To rule out the effects of DNA polymerase-induced errors during amplification, sequencing errors and assembly artifacts, a similar analysis was performed using sequences obtained using the *ERVWE1* encoding plasmid as template for PCR (gift from Dr. F. Mallet). The number of non-synonymous (K_a_) and synonymous (K_s_) substitutions per site (averaged over the sequenced length) were estimated for all MS and non-MS sequences analyzed using modified LWL method in Kaks calculator software [41].

### Western blotting

Fetal and adult brain samples were lyzed using RIPA buffer. Total protein concentrations were quantified with Bio-Rad protein assay kit (Bio-Rad Life Sciences Group, Hercules, CA) and 80 µg of protein per sample/treatment was electrophoresed on a 10% SDS-PAGE gel (sodium dodecyl sulfate-polyacrylamide gel) under reducing conditions. Proteins were transferred to a PVDF membrane (BioRad) and blocked for 3 hours in 5% milk in TBST (20 mM Tris–HCl, pH 7.6, 0.15 M NaCl and 0.1% Tween-20). The membrane was incubated overnight at 4°C with rabbit polyclonal anti-Syncytin-1 primary Ab (1∶300), generated in house using a peptide sequence TGMSDGGGVQDQAREKHV and subsequently with horseradish peroxidase (HRP)-conjugated donkey anti-rabbit secondary Ab (1∶25,000) for 2 h at room temperature. Specific immunoreactive proteins were visualized using the BM Chemiluminescence Western Blotting Substrate (POD) reagent (Roche Diagnostics, Laval, QC) followed by quantification with the Quantity One software (BioRad).

### Bioinformatic analyses

The sequence tags derived from deep sequencing of fetal and adult (healthy) brain samples were analyzed for the abundance of different host immune genes. Bioinformatic analysis was performed to glean insight into the functional aspects of host genes with expression levels highly correlated with those of *ERVWE1*. All genes, which passed the filtering criteria (≥2 tags detected per sample) and showed a high degree of correlation with respect to HERV-W *env* transcript levels (Pearson r^2^≥0.75), were analyzed for overrepresented functional classes by Gene Ontology (GO) term examination using DAVID (http://david.abcc.ncifcrf.gov).

### Statistical analyses

Parametric and nonparametric statistical tests were performed using GraphPad Prism version 4.0 (GraphPad Software, San Diego, CA, USA). Chi-square test was used for comparison of categorical variables between human fetal neuron, astrocytes and microglia. *p* values of <0.05 were considered significant.

## Supporting Information

Figure S1 **Comparison of HERV-W** ***env*** **sequences.** **(A)**. The 10 homologous genomic regions with the highest BLAT scores over the entire length of the ERVWE1 ORF were identified and aligned using Clustal W. Shown are the truncated representative regions of the cloned HERV-W sequences used for primer design. The boxed region represents the most heterogenous sequence region among the predicted sequences. **(B)** Specific chromosomal loci distribution of transcriptionally active HERV-W *env* sequences from human placenta; the nucleotide sequence alignment of HERV-W *env* (ERVWE1) and one of its putative paralogs located on chromosome 14 with three representative sequences derived from human placenta. The sequences are distinguishable by several nucleotide differences; the residues, which are conserved in the sequences, have a white background while the black background shows lack of sequence conservation among different sequences. While the Plac04 was similar to ERVWE1, but different from that of paralog sequence at chromosome loci 17 and Plac01 and Plac05 sequences. The shaded nucleotides are conserved among different paralogs, which differentiates them from HERV-W *env* sequence located on the chromosome location 7q21.2. Based on this analysis, chromosomal loci were assigned the present cloned sequences using BLASTN.
(EPS)

Figure S2 Phylogenetic analyses of selected sequences from non-diseased brain specimens. The tree was rooted to MSRV as an out group gene sequence.
(EPS)

Table S1 Quantitative analyses of MS and non-MS sequences.
(PDF)

Table S2 PCR and real-time oligonucleotide primer sequences.
(PDF)
