# Supplementary material for: Age- and Disease-Dependent HERV-W Envelope Allelic Variation in Brain: Association with Neuroimmune Gene Expression
Source: PLoS One. 2011 Apr 29;6(4):e19176. doi: 10.1371/journal.pone.0019176 (PMC3084769; doi:10.1371/journal.pone.0019176)
Supplement: Table S1 — Quantitative analyses of MS and non-MS sequences. (PDF) [file pone.0019176.s003.pdf]

Table ST1: Quantitative analyses of MS and non-MS sequences.

Sample ID	Number of sequences	Mean sequence diversity /sample[a]	Mean diversity within sample	Ka/Ks per sample
MS01	10	0.409	0.002	0.1292
MS02	6	0.410	0.001	0.0844
MS03	10	0.606	0.004	0.0910
MS04	10	0.693	0.006	0.5490
MS05	10	0.834	0.004	0.1374
MS06	8	0.836	0.005	0.1276
MS07	9	0.490	0.002	0.1325
MS08	9	0.962	0.005	0.1290
non-MS01	8	0.450	0.001	0.0827
non-MS02	10	0.861	0.006	0.0770
non-MS03	10	0.442	0.001	0.1219
non-MS04	9	0.459	0.002	0.0887
non-MS05	10	0.508	0.001	0.1265
non-MS06	10	0.384	0.002	0.1277
non-MS08	10	0.378	0.000	0.1190

[a] **Relative to *ERVWE1***
