# Supplementary material for: Age- and Disease-Dependent HERV-W Envelope Allelic Variation in Brain: Association with Neuroimmune Gene Expression
Source: PLoS One. 2011 Apr 29;6(4):e19176. doi: 10.1371/journal.pone.0019176 (PMC3084769; doi:10.1371/journal.pone.0019176)
Supplement: Table S2 — PCR and real-time oligonucleotide primer sequences. (PDF) [file pone.0019176.s004.pdf]

Table ST2: PCR and real-time oligonucleotide primer sequences.

Description	Primer Name	Nucleotide Sequence
HERV-W env -1st step nested PCR primers	Sense1	5’- GATGGATCCAAAACTACATGAAACCT -3’
	Anti-sense1	5’- CGTGACTCGAGCTGCTTCCTGC - 3’
HERV-W env -2nd step nested PCR primers	Sense2	5’- GATGGATCCAGCCTATTTAATACCAC - 3’
	Anti-sense2	5’ - CGTGACTCGAGAGTTAAGTTGATCTTGCAA - 3’
HERV-W env - real time PCR primers	Sense	5’ - TGCCCCATCGTATAGGAGTCT - 3’
	Anti-sense	5’ - CATGTACCCGGGTGAGTTGG - 3’
GAPDH – real time PCR primers	Sense	5’ - AGCCTTCTCCATGGTGGTGAAGAC - 3’
	Anti-sense	5’ - CGGAGTCAACGGATTTGGTCG - 3’
TGF-Beta[a]	Sense	5’ - TTCAACACATCAGAGCTCCGA - 3’
	Anti-sense	5’ - GGAGAGCAACACGGGTTCAG - 3’
CD3	Sense	5’ - GATGCAGTCGGGCACTCACT - 3’
	Anti-sense	5’ - CATTACCATCTTGCCCCCAA - 3’
HLA-DRA[b]	Sense	5’ - GGACAAAGCCAACCTGGAAA - 3’
	Anti-sense	5’ - AGGACGTTGGGCTCTCTCAG - 3’
IL-12p35[c]	Sense	5’ - AGCCTCCTCCTTGTCGCTACC - 3’
	Anti-sense	5’ - GCCTCCACTGTGCTGGTTTTATC - 3’
Granzyme A	Sense	5’ - ACTCTGAGAGAAGTCAATATCACC- 3’
	Anti-sense	5’ - AGGGCTTCCAGAATCTCCAT - 3’
CD11c[d]	Sense	5’ - ACCACAAGCAGTAGCTCCTTC - 3’
	Anti-sense	5’ - AAGTAGGAGCCGATCTGAGTC - 3’
GFAP	Sense	5’- GGACATCGAGATCGCCACCTACAG - 3’
	Anti-sense	5’ - CTCACCATCCCGCATCTCCACAGT - 3’
IL-10[e]	Sense	5’ - AATAAGGTTTCTCAAGGGGCT - 3’
	Anti-sense	5’ - AGAACCAAGACCCAGACATCAA - 3’
IL-23p19[f]	Sense	5’ - GAGCCTTCTCTGCTCCCTGAT - 3’
	Anti-sense	5’ - AGTTGGCTGAGGCCCAGTAG - 3’
CD8b[g]	Sense	5’ - TACTTCTGCATGATCGTCGG - 3’
	Anti-sense	5’ - ACCGGCACACTCTCTTCTTG - 3’
HLA-DQA1[b]	Sense	5’ - GGTGTAAACTTGTACCAGT - 3’
	Anti-sense	5’ - GGAGACTTGGAAAACACT - 3’

[a] Schaub, B., et al., 2006. Neonatal immune responses to TLR2 stimulation: influence of maternal atopy on Foxp3 and IL-10 expression. Respir Res. 7, 40.

[b] Malanga, D., et al., 2007. The active translation of MHCII mRNA during dendritic cells maturation supplies new molecules to the cell surface pool. Cell Immunol. 246, 75-80.

[c] Durali, D., et al., 2003. In human B cells, IL-12 triggers a cascade of molecular events similar to Th1 commitment. Blood. 102, 4084-9.

[d] Lee, T. J., et al., 2006. Se-methylselenocysteine enhances PMA-mediated CD11c expression via phospholipase D1 activation in U937 cells. Immunobiology. 211, 369-76.

[e] Pattyn, F., et al., 2006. RTPrimerDB: the real-time PCR primer and probe database, major update 2006. Nucl. Acids Res. 34, D684-688.

[f] Vaknin-Dembinsky, A., et al., 2006. IL-23 Is Increased in Dendritic Cells in Multiple Sclerosis and Down-Regulation of IL-23 by Antisense Oligos Increases Dendritic Cell IL-10 Production. J Immunol. 176, 7768-7774.

[g] Cui, W., et al., 2007. qPrimerDepot: a primer database for quantitative real time PCR. Nucleic Acids Res. 35, D805-9.
